# Reducing analogue trauma symptoms by computerized reappraisal training – Considering a cognitive prophylaxis?

**DOI:** 10.1016/j.jbtep.2013.01.003

**Published:** 2013-09

**Authors:** Marcella L. Woud, Peggy Postma, Emily A. Holmes, Bundy Mackintosh

**Affiliations:** a Behavioural Science Institute, Radboud University Nijmegen, Montessorilaan 3, Room A07.03, 6525 HR Nijmegen, The Netherlands; b Hertfordshire Partnership NHS Foundation Trust, Hertford, UK; c MRC Cognition and Brain Science Unit, Cambridge, UK; d Department of Psychology, University of Essex, Essex, UK

**Keywords:** Posttraumatic stress disorder (PTSD), Reappraisal training, Cognitive bias modification (CBM), Trauma film, Intrusions

## Abstract

**Background and objectives:**

Distressing intrusions are a hallmark of posttraumatic stress disorder (PTSD). Dysfunctional appraisal of these symptoms may exacerbate the disorder, and conversely may lead to further intrusive memories. This raises the intriguing possibility that learning to ‘reappraise’ potential symptoms more functionally may protect against such symptoms. Woud, Holmes, Postma, Dalgleish, and Mackintosh (2012) found that ‘reappraisal training’ when delivered *after* an analogue stressful event reduced later intrusive memories and other posttraumatic symptoms. The present study aimed to investigate whether reappraisal training administered *before* a stressful event is also beneficial.

**Methods:**

Participants first received positive or negative reappraisal training (CBM-App training) using a series of scripted vignettes. Subsequently, participants were exposed to a film with traumatic content. Effects of the CBM-App training procedure were assessed via three distinct outcome measures, namely: (a) post-training appraisals of novel ambiguous vignettes, (b) change scores on the Post Traumatic Cognitions Inventory (PTCI), and (c) intrusive symptom diary.

**Results:**

CBM-App training successfully induced training-congruent appraisal styles. Moreover, those trained positively reported less distress arising from their intrusive memories of the trauma film during the subsequent week than those trained negatively. However, the induced appraisal bias only partly affected PTCI scores.

**Limitations:**

Participants used their own negative event as a reference for the PTCI assessments. The events may have differed regarding their emotional impact. There was no control group.

**Conclusions:**

CBM-App training has also some beneficial effects when applied before a stressful event and may serve as a cognitive prophylaxis against trauma-related symptomatology.

## Highlights

► Reappraisal training (i.e., CBM-App training) can serve as a cognitive prophylaxis. ► CBM-App training successfully induced training congruent appraisal styles. ► Participants in positive CBM-App training group reported less intrusion distress.

## 1 Introduction

Posttraumatic stress disorder (PTSD) is a distressing psychological reaction to a traumatic event (Diagnostic and Statistical Manual for Mental Disorders; DSM-IV-TR, 2000). Distressing intrusions, i.e., the involuntary re-experiencing of the trauma, are a hallmark symptom. According to cognitive models of PTSD (e.g. Dalgleish, 2004; Ehlers & Clark, 2000), a maintaining factor is the maladaptive appraisals associated with having intrusions (“Having these flashbacks means I'm going mad”), as they may be interpreted as a sign of permanent psychological damage. Furthermore, models stress the role of maladaptive appraisals when developing PTSD. Prospective studies such as executed by Bryant and Guthrie (2005, 2007) support this. In their studies, trainee fire-fighters completed the Post Traumatic Cognitions Inventory (PTCI; Foa, Ehlers, Clark, Tolin, & Orsillo, 1999), which measures appraisals following trauma, before they were exposed to stressful situations. Fire-fighters were assessed for PTSD symptomatology after six months (Bryant & Guthrie, 2005) and after four years (Bryant & Guthrie, 2007) of fire-fighting-duty. Results showed that a baseline tendency to engage in maladaptive appraisals predicted subsequent PTSD, particularly scores on the PTCI-Self subscale.

This raises the intriguing possibility that learning to ‘reappraise’ may be of importance in PTSD. There is an emerging body of research investigating the beneficial effects of reappraisal, for example as an emotion-regulation-strategy in healthy participants (Gross, 2002), in clinical contexts such as depression (e.g. Lang, Moulds, & Holmes, 2009) and in analogue posttraumatic-stress (e.g. Woud et al., 2012). To illustrate, Woud et al. trained participants to engage in positive or negative appraisal styles after having been exposed to distressing films. The computerized training targeted self-efficacy beliefs and reappraisals of secondary emotions, i.e., emotions in response to the emotional reactions elicited by the films. Procedures were based on methods developed within the Cognitive Bias Modification (CBM) framework (cf. Koster, Fox, & MacLeod, 2009). Results demonstrated that the CBM-Appraisal (CBM-App) training successfully induced training congruent appraisal styles. Moreover, compared to those trained negatively, those trained positively reported fewer intrusive memories of the film, and had lower scores on the PTCI (Foa et al., 1999) as well as on the Impact of Event Scale-Revised (IES-R; Weiss & Marmer, 1997) at one-week follow-up (the IES-R is a widely-used clinical measure of posttraumatic stress).

In sum, results support that reappraisal training does have therapeutic effects when applied *after* the traumatic event. However, could it also have prophylactic effects when applied *before* the traumatic event? The current study set out to explore this. Therefore, we adapted the procedure of Woud et al. (2012) such that participants first completed positive or negative CBM-App training and then watched the distressing films. We predicted that CBM-App training would successfully induce either an adaptive or maladaptive appraisal style (depending on the training condition). This was tested via post-training appraisals of novel ambiguous vignettes and via PTCI scores post-film and at one-week follow-up. Second, we predicted that those trained positively would report fewer intrusions of the stressor films and less intrusion distress than those trained negatively.

## 2 Methods

### 2.1 Participants

Out of the panel of community based volunteers of the MRC Cognition and Brain Sciences Unit, 54 participants were recruited. Selection criteria were fluent written English ability, no reported psychological problems or past traumatic experiences, and sub-clinical scores on the State-Trait Anxiety Inventory (STAI; Spielberger, Gorsuch, Lushene, Vagg, & Jacobs, 1983) and on the Beck Depression Inventory (BDI-II; Beck, Steer, & Brown, 1996), i.e., at or below 40 and 13, respectively (Harrison & Turpin, 2003).

### 2.2 Materials

#### 2.2.1 Self report measures

State-Trait anxiety was assessed via the State and Trait Anxiety Inventory (STAI-S, STAI-T; Spielberger et al., 1983). Depressive symptoms were measured via the Beck Depression Inventory-II (BDI-II; Beck et al., 1996). The Posttraumatic Cognitions Inventory (PTCI; Foa et al., 1999) was used to assess appraisals surrounding distressing and/or traumatic experiences, including all three subscales (negative cognitions about Self, the World and Self-Blame).

#### 2.2.2 Mood rating

Four mood states were assessed (happiness, depression, anger, anxiety) using an 11-point Likert scale ranging from 0 = “not at all” to 10 = “extremely” (Davies & Clark, 1998). Scores were averaged (with reversed happiness scores) to provide a single mood index.

#### 2.2.3 Reappraisal training (CBM-App)

##### 2.2.3.1 Training phase

A detailed description of the applied CBM-App training procedure can be found in Woud et al. (2012). Participants were presented with a series of ambiguous, reappraisal-related scripted vignettes (henceforth called ‘scripts’) that ended in a to-be-completed word fragment. Participants had to complete the word fragment by typing in its first missing letter. These words then produced an outcome which was consistent with either an adaptive or maladaptive appraisal of the ambiguous script.

Scripts were based on items of the PTCI Self subscale. For example, “trusting oneself to act appropriately in future” was adapted as follows: ‘*In a crisis, I predict my responses will be h-lpf-l / u-el-ss’* (resolved as ‘*helpful*’ in the positive or ‘*useless*’ in the negative CBM-App condition). Just under half of these scripts were followed by a question to test ongoing comprehension. The training comprised 72 training and 8 emotionally neutral filler scripts (presented in blocks of 10). Blocks were presented in the same order for each participant but the sentences' order within each block was individually randomized.

##### 2.2.3.2 Measuring induced reappraisal bias

The training's success was assessed via a two-phase-procedure (Mathews & Mackintosh, 2000). During the encoding-phase, participants read 10 novel ambiguous scripts in random order. Scripts started with a title and, unlike the former training items, remained ambiguous. Participants were asked to imagine themselves vividly in the described situation (assessed via a 10-point Likert scale). In the recognition-phase, the 10 encoding-phase titles were presented again, followed by a set of 4 related sentences. By means of a 4-point Likert scale, participants rated how close in meaning each sentence was to the original script of that title. There were two *target* sentences, representing a possible positive and negative interpretation of the original script, and there were two *foil* sentences, representing a general positive and negative meaning that did not resolve the script's ambiguity (see Appendix A).

#### 2.2.4 Stressor film

The 20-min distressing film was a compilation of 1–3 min clips that have been used in Woud et al. (2012), displaying contents such as footages from the 9/11 World Trade Center terrorist attack or motor vehicle accidents. Participants had to view the film “as if they were there, a bystander at the scene of the events”, and to pay attention to the film as later there may be questions about film content. By means of a 10-point Likert scale participants rated their attention paid to the film (see Holmes & Steel, 2004).

#### 2.2.5 Intrusion diary

To record film-related intrusions as well as intrusion distress, participants received a 7-day-diary (see Holmes, Brewin, & Hennessy, 2004; Holmes, James, Coode-Bate, & Deeprose, 2009). Intrusions were defined as “any memory of the film (or part of the film) that appeared apparently spontaneously in your mind. Do not include any memories of the film that you deliberately or consciously bring to mind”. Participants were instructed to record all intrusions immediately after they occurred (whenever possible) and to check their diary each day at a fixed time point to make sure that it was up-to-date.

Explanations were given about the types of intrusions: “What goes through our minds can either take the form of words and phrases (‘verbal thoughts’), or it can be like mental images. Although mental images often take the form of pictures they can actually include any of the five senses, so you can imagine sounds or smells too.” Participants were asked to specify whether their intrusion was a thought or image or a combination of both, and what its exact contents was.

#### 2.2.6 Diary compliance rating

Participants rated their diary completion in comparison with the statement ‘I have often forgotten (or have been unable) to record my intrusive thoughts or images in the diary’ on a scale ranging from 0 (*not at all true of me*) to 10 (*completely true of me*) (Davies & Clark, 1998).

### 2.3 Procedure

After informed consent, participants completed the STAI-T and BDI-II. Based on the STAI-T and BDI-II selection criteria, twelve participants were excluded. Then, STAI-S, PTCI and the first mood rating followed. As PTCI-responses are anchored to a specific traumatic event, participants were asked to use an own negative event as a reference (see Bryant & Guthrie, 2005, 2007). This instruction was used during all three PTCI assessments. The CBM-App training was presented next, with participants randomly allocated to either positive or negative CBM-App. Again, participants used an own negative event as a reference. After that, the encoding-recognition phase followed. Participants then watched the stressor film and completed the attention-to-film-assessment and the second mood and PTCI rating, respectively. Finally, the 7-day-diary and PTCI were distributed. One week later participants were contacted via telephone to discuss their diary entries. They were debriefed and thanked and if they had not already done so, prompted to return the diary, the PTCI and diary compliance rating using a pre-paid envelope that had been supplied.

## 3 Results

### 3.1 Participant characteristics

Data of 7 participants were excluded. Two participants failed to submit the diaries, the remaining 5 participants were identified as multivariate outliers on BDI, intrusion frequency and distress (2 in negative and 3 in positive CBM-App condition) by calculating Mahalanobis distance with the criterion for outliers set at *p* = .05 (Tabachnick & Fidell, 1996). The final sample included 47 participants (31 women, *M*_age_ = 29.06, *SD* = 10.02). The two CBM-App Training Groups did not differ on age, STAI-S, STAI-T, BDI and mood, nor on attention paid to the film or diary compliance (see for means, standard deviations and significances Table 1).

### 3.2 Effects of film on mood

A mixed-model ANOVA with Time (pre versus post film mood) as a within-subjects factor and CBM-App Training Group (negative versus positive) as a between-subjects factor showed a significant main effect of Time, *F*(1,45) = 29.10, *p* < .001, $\eta_{p}^{2}$ = .39, though no significant main effect of Training Group, *F*(1,45) = .02, *p* = . 88. Importantly, there was no significant Time × CBM-App Training Group interaction, *F*(1,45) = 1.19, *p* = .28, confirming that the film had a negative impact on mood across both groups (see Table 1 for means and standard deviations).

### 3.3 Manipulation check: assessment bias index and PTCI scores

The raw data from the recognition-phase were converted into a bias index by subtracting the mean ratings for negative targets from those of positive targets. Hence, a positive bias index indicated that positive targets were rated closer in meaning to the ambiguous sentences than negative targets, and vice versa for a negative bias index. Analyses revealed a significant difference in bias index between the two CBM-App training groups, *t*(45) = 14.09, *p* < .001, *d* = 4.10, with each group yielding a mean bias significantly different from zero in the anticipated direction, positive CBM-App group: *t*(21) = 11.51, *p* < .001, *d* = 2.44 (*M* = 1.78, *SD* = .73), negative CBM-App group: *t*(24) = 8.91, *p* < .001, *d* = 1.78 (*M* = −1.58, *SD* = .89).

PTCI changes were explored via a mixed model ANOVA. There was no significant main effect of CBM-App Training Group, *F*(1,45) = .12, *p* = .73, though a significant main effect of Time, *F*(2,44) = 8.42, *p* < .01, $\eta_{p}^{2}$ = .28, and a significant interaction between Time and Training Group, *F*(2,44) = 3.17, *p* = .052, $\eta_{p}^{2}$ = .13. Investigating this interaction more thoroughly, analyses revealed a significant improvement (lower scores) when comparing baseline versus one-week follow-up in the positive CBM-App group, *t*(21) = 2.93, *p* < .01, *d* = .30. No improvement was found when comparing baseline versus post-film assessment, *t*(21) = .70, *p* = .49. Scores in the negative CBM-App group became significantly worse (higher scores) when comparing baseline versus post-film assessment, *t*(24) = 3.54, *p* < .01, *d* = .23. However, the baseline versus one-week follow-up comparison was not significant, *t*(24) = .88, *p* = .39.

### 3.4 Film intrusions over one week

Intrusions were experienced by 80% of participants in the negative CBM-App group and 77.3% of participants in the positive negative CBM-App group with no significant group differences, *χ*^2^(1) = .05, *p* = .82. The analysis of intrusion distress was necessarily confined to the 78.7% of those who had experienced intrusions. There was a significant difference between CBM-App training groups *t*(35) = 2.23, *p* = .03, *d* = .79, with less distress for those trained positively (positive: *M* = 12.23, *SD* = 9.25, negative: *M* = 22.74, *SD* = 17.46). However, there was no significant difference between the two groups regarding the frequency of intrusions, *t*(45) = .26, *p* = .79 (positive: *M* = 7.14, *SD* = 8.08; negative: *M* = 6.56, *SD* = 6.95).

## 4 Discussion

CBM-App training successfully induced training-congruent appraisal styles: Participants trained positively appraised novel ambiguous vignettes in a more adaptive manner than participants trained negatively. Regarding the PTCI, those trained positively did not improve immediately after the training phase (i.e., baseline versus post film), though did improve when comparing baseline versus one-week follow-up. PTCI scores of those trained negatively became worse post-film, but no difference was found between baseline versus one-week follow-up. Results of the 7-day-diary showed that those trained positively experienced less intrusion distress than those trained negatively. No significant difference was found regarding the frequency of film-related intrusions.

The present findings corroborate the significant role of reappraisal in PTSD, showing that CBM-App training can have some preventative function in analogue traumatic stress. Results support assumptions of cognitive models of PTSD (e.g. Ehlers & Clark, 2000) and provide an interesting extension of the CBM-training literature. Moreover, these data could encourage some first, careful clinical applications for assisting individuals who may encounter traumatic events such as policeman or fire-fighters. However, they also demonstrate the boundaries of such CBM procedures: PTCI scores were only partly affected, and the change in scores might be to some extent driven by the non-significant baseline differences between the two training groups. Finally, CBM-App training only reduced intrusion distress. The prospective nature of this study might explain this. Maybe, participants were less engaged during the training, as nothing actively distressed them at that point. Moreover, participants' STAI and BDI scores were low (compared to those reported in Woud et al., 2012), and this also may have influenced the trainings' effect.

The present findings are not without limitations. During the PTCI measurements, participants had to think of their own stressful event. However, we do not know anything of the emotional impact of the event and whether participants were compliant in general. Hence, although this procedure has been applied successfully before (e.g. Bryant & Guthrie, 2005, 2007), this is may be an element in our procedure that may have produced noise. Second, our design did not include a control group receiving neutral CBM-App training. Nevertheless, results showed training-congruent biases that differed from zero.

To conclude, CBM-App training also has beneficial effects on trauma-related symptomatology when applied before a stressful event. These findings clearly advance our understanding of the role of reappraisal in PTSD: CBM-App training not only has a therapeutic (Woud et al., 2012), but also a *prophylactic* effect. Future research now has to target the potential underlying mechanisms of CBM-App training in order to crystallize the processes that produced the effects and to optimize its application.

## Figures and Tables

**Table:** 

Title	“Aftermath of events.”
Encoding script	“People come to terms with the aftermath of these types of events in many different ways. My reactions are very indicative of the way I seem to be dealing with it.”
Recognition sentences	
Negative target	“People come to terms with the aftermath of these types of events in many different ways, but my reactions mean my coping skills are poor.”
Positive target	“People come to terms with the aftermath of these types of events in many different ways, but my reactions mean my coping skills are healthy.”
Negative foil	“People come to terms with the aftermath of these types of events in many different ways, but my reactions mean my coping skills are suspicious.”
Positive foil	“People come to terms with the aftermath of these types of events in many different ways, but my reactions mean my coping skills are trustworthy.”

**Table 1:** Demographics & self report data, mood, PTCI and diary data.

Measure	Negative CBM-App (*n* = 25, 16 female)		Positive CBM-App (*n* = 22, 15 female)		*t*(45)	*p*
	*M*	*SD*	*M*	*SD*		
Age	29.88	10.16	28.13	10.02	.59	.56
STAI-S	26.52	5.59	27.91	6.48	.79	.43
STAI-T	29.84	5.73	31.23	6.15	.80	.43
BDI-II	2.56	2.22	3.95	3.42	1.68	.10
Baseline mood	1.03	.73	1.35	.79	1.46	.15
Post-film mood	2.64	1.93	2.42	1.79	.40	.69
Attention to film	9.40	.76	9.50	.60	.50	.62
Diary compliance	.72	1.17	.73	.98	.02	.98
PTCI time 1	84.44	35.18	95.18	42.38	.95	.35
PTCI time 2	93.60	43.78	92.31	39.31	.11	.92
PTCI time 3	80.80	37.60	83.00	39.30	.20	.85

*Note*. STAI-S/T = Spielberger State-Trait Anxiety Inventory-State/Trait version; BDI-II = Beck Depression Inventory-II; PTCI = Posttraumatic Cognitions Inventory.

## Appendix A

Examples of stimuli used to measure induced reappraisal bias including Title, Ambiguous Encoding Script, and Recognition Sentences used to assess induced bias following bias modification. Each recognition sentence was rated for similarity to the original titled script using a four point scale ranging from 1 (very different in meaning) to 4 (very similar in meaning).
